# Digitally Designed Bone Grafts for Alveolar Defects: A Scoping Review of CBCT-Based CAD/CAM Workflows

**DOI:** 10.3390/jfb16090310

**Published:** 2025-08-28

**Authors:** Francesco Puleio, Giuseppe Lo Giudice, Gaetano Marenzi, Rosaria Bucci, Riccardo Nucera, Roberto Lo Giudice

**Affiliations:** 1 Department of Biomedical and Dental Sciences and Morphofunctional Imaging, University of Messina, 98122 Messina, Italy; roberto.logiudice@unime.it; 2 Department of Clinical and Experimental Medicine, University of Messina, 98122 Messina, Italy; giuseppe.logiudice@unime.it; 3 Department of Neurosciences, Reproductive Sciences and Oral Sciences, Postgraduate School of Oral Surgery Division, University of Naples Federico II, 80131 Naples, Italy; gaetano.marenzi@unina.it; 4 Department of Neurosciences, Reproductive Sciences and Oral Sciences, Postgraduate School of Orthodontics and Temporomandibular Disorder Clinic, University of Naples Federico II, 80131 Naples, Italy; rosaria.bucci@unina.it; 5 Department of Biomedical and Dental Sciences and Morphofunctional Imaging, Section of Orthodontics, School of Dentistry, University of Messina, 98100 Messina, Italy; riccardo.nucera@unime.it

**Keywords:** computer-aided design and manufacturing (CAD/CAM), custom bone graft, bone regeneration, alveolar ridge augmentation, scoping review, digital workflow, cone-beam CT

## Abstract

This scoping review aimed to systematically map the literature on digital workflows for the design and fabrication of customized bone grafts in oral and maxillofacial surgery. The review focused on the integration of cone-beam computed tomography (CBCT), computer-aided design (CAD), and computer-aided manufacturing (CAM) techniques for the production of personalized bone blocks. A systematic search of PubMed, Web of Science, and Ovid MEDLINE identified 151 records published between 2015 and 2025; after duplicate removal, screening, and full-text assessment, 16 articles were included. Six additional seminal studies published before 2015 were considered through manual search to provide historical background. The included studies consisted of case reports, case series, prospective clinical investigations, and preclinical experiments. Customization strategies involved synthetic hydroxyapatite scaffolds, CAD/CAM-milled allogeneic blocks, xenogeneic blocks, and digitally guided autogenous grafts. Four studies provided direct clinical documentation of customized CAD/CAM bone blocks, while the others offered complementary evidence on digital design, scaffold adaptation, or preclinical validation. Outcomes included graft adaptation, volumetric stability, implant survival, and limited histological analyses. Despite promising short-term results, no study has yet described the complete clinical workflow from CBCT acquisition to milling and implantation of a biological autologous or xenogeneic block in humans. This review underscores both the feasibility and the limitations of current approaches, highlighting the absence of fully validated digital-to-biological protocols as the main gap to be addressed in future research.

## 1. Introduction

The regeneration of bone defects in the maxillary bones represents a common yet complex clinical challenge in oral implantology and maxillofacial surgery. Maxillary atrophy may result from tooth loss, infections, trauma, surgical resections, or chronic pathological conditions, leading to a tridimensional reduction in the available bone volume for implant-supported rehabilitation. In particular, the alveolar ridge tends to undergo resorption both vertically and horizontally, with greater compromise of the buccal wall, often requiring reconstructive procedures prior to implant placement [1,2].

Currently, the most commonly used techniques for bone regeneration include the following:

- The use of autologous block grafts, considered the gold standard due to their osteogenic, osteoinductive, and osteoconductive properties. These grafts are typically harvested from intraoral sites (mandibular symphysis, ascending ramus, tuberosity) or extraoral sites (iliac crest), but they present several limitations: donor site morbidity, increased surgical time, postoperative pain, risk of paresthesia, intraoperative shaping difficulties, and unpredictable volumetric resorption [3,4,5,6].
- The use of particulate bone grafts (autologous, homologous, xenogeneic, or synthetic), combined with resorbable or non-resorbable membranes for guided bone regeneration (GBR). Although GBR is well-validated and widely applied, it requires strict stability of the blood clot, carries a high risk of membrane exposure, involves prolonged healing times, and may result in insufficient quantitative or qualitative bone regeneration [7,8,9].
- The use of preformed or 3D-printed grafting materials, such as synthetic scaffolds (e.g., hydroxyapatite or β-TCP), or standardized allogenic/xenogenic blocks. These approaches may also suffer from mismatch between graft and actual defect, difficulties in stabilization, poor anatomical adaptation, and variability in regenerative outcomes [10,11,12,13].

Although effective, these techniques exhibit limitations related to poor adaptation accuracy, surgical complexity, material variability, and the unpredictability of clinical outcomes. These issues highlight the need for more predictable and personalized approaches based on digital technologies.

In this context, the integration of three-dimensional imaging (CBCT), CAD modeling, and subtractive manufacturing techniques (CAM) has paved the way for the development of customized bone grafts. Several authors have described the use of tailored scaffolds obtained by milling hydroxyapatite blocks, synthetic materials, or standardized allogenic grafts, shaped from STL files derived from patient-specific CBCT data [14,15,16]. These customized grafts offer improved defect adaptation, better primary stability, and reduced surgical time [14,15,16].

However, to date, no studies have reported the clinical milling and implantation of a customized biological graft (autologous or xenogeneic), such as natural equine or porcine bone blocks, derived from a patient-specific CBCT scan, including the segmentation of the defect and production of a graft perfectly shaped to the patient’s anatomical model. Although several proof-of-concept studies on animal models or using synthetic materials are available, and some clinical cases have been reported using milled allogenic blocks, there is still no clinical documentation of custom-shaped biological grafts manufactured via subtractive milling from human CBCT datasets.

Therefore, a comprehensive mapping of the current literature is needed to evaluate the state of available technologies and identify gaps in the CBCT → STL → CAD → CAM → biological graft workflow.

The aim of this scoping review is to systematically map and analyze the existing scientific literature on the use of CBCT-based, digitally designed, and milled or printed bone grafts for the personalized regeneration of maxillary bone defects.

The specific objectives are as follows:

- To identify and describe digital workflows used in the planning and design of custom bone grafts for maxillary bone regeneration.
- To categorize the modeling and manufacturing techniques (e.g., CAD/CAM milling, 3D printing) employed to fabricate patient-specific bone grafts.
- To evaluate the types of bone graft materials (autologous, allogenic, xenogeneic, synthetic) utilized in clinical and preclinical applications.
- To summarize reported clinical, radiographic, histological, volumetric, and implant-related outcomes associated with the use of personalized bone grafts.

## 2. Materials and Methods

### 2.1. Protocols

This scoping review was conducted in accordance with the PRISMA-ScR guidelines (Preferred Reporting Items for Systematic reviews and Meta-Analyses extension for Scoping Reviews), which were developed to enhance transparency and completeness in the reporting of reviews with exploratory and descriptive aims, particularly those focused on mapping the current state of knowledge on a given research topic [17].

This scoping review protocol was registered on the Open Science Framework (OSF) to ensure transparency and methodological reproducibility. The full protocol is publicly accessible at DOI: https://doi.org/10.17605/OSF.IO/7Z2PT.

The protocol was structured according to the PCC framework (Population–Concept–Context), which allows for the inclusion of a broad range of study designs and methodological approaches:

- Population: patients presenting with bone defects in the mandible or maxilla;
- Concept: application of digital workflows for the design and fabrication of customized bone grafts using 3D imaging, STL/CAD modeling, and CAM milling;
- Context: bone regeneration in oral surgery, maxillofacial surgery, and implantology.

The objective was to explore the existing literature to determine whether custom-milled biological bone blocks (autologous or xenogeneic) have already been used in clinical or preclinical models based on patient-specific CBCT scans, and to assess which technologies are currently available or under development along the CBCT → STL → CAD → CAM → graft pathway.

### 2.2. Search Strategy

The literature search was conducted on 9 July 2025, using two electronic databases: PubMed (MEDLINE), the Web of Science Core Collection and Ovid MEDLINE. Articles published between 1 January 2015, and 30 June 2025, were considered. Only articles written in English with full-text availability were included. Conference abstracts, letters to the editor, and non-peer-reviewed articles were excluded.

For each database, a structured search strategy was developed using Boolean operators and adapted to the specific syntax of each search engine. The exact search strings used are reported below:

- PubMed: (“Bone Regeneration” [MeSH Terms] OR “Ridge Augmentation” OR “Bone Graft” OR “Alveolar Ridge Augmentation”) AND (“CAD/CAM” OR “Computer-Aided Design” OR “Custom Block” OR “Milling”) AND (“CBCT” OR “Cone Beam Computed Tomography” OR “STL File” OR “3D Printing”)
- Web of Science: TS = (“Bone Regeneration” OR “Ridge Augmentation” OR “Bone Graft” OR “Alveolar Ridge Augmentation”) AND TS = (“CAD/CAM” OR “Computer-Aided Design” OR “Custom Block” OR “Milling”) AND TS = (“CBCT” OR “Cone Beam Computed Tomography” OR “STL File” OR “3D Printing”)
- Ovid MEDLINE: (bone regeneration or ridge augmentation or bone graft* or alveolar ridge augmentation) .mp. AND (cad cam or computer aided design or computer aided manufacturing or custom* block* or custom* graft* or custom* scaffold* or milling or 3d print*) .mp. AND (cbct or cone beam or stl or stereolithograph* or 3d model*) .mp. AND english.lg. AND yr = “2015-Current”

All results were exported and managed using bibliographic screening software (EndNote v.21.5). After removal of duplicates, titles and abstracts were independently screened by two reviewers. Potentially relevant articles were selected for full-text review. Any disagreements were resolved through discussion or with the involvement of a third reviewer.

**Figure 1:**
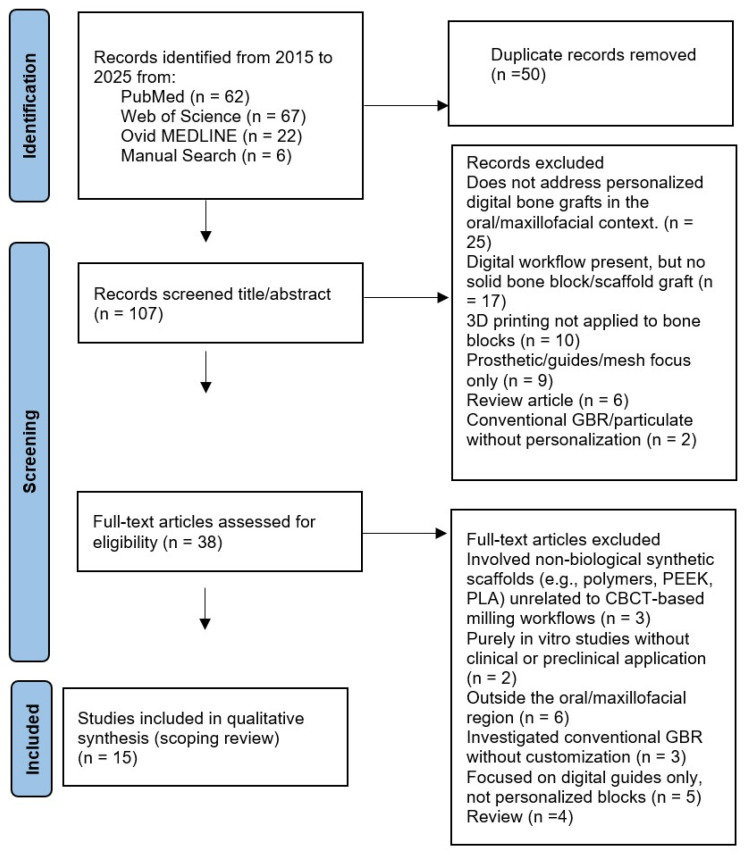
PRISMA flow chart.

The selection process is illustrated in the PRISMA-ScR flow chart (Figure 1), which documents the number of articles retrieved from each database, the duplicates removed, the exclusions made based on language, date, methodology, or topic, and the final number of studies included in the qualitative synthesis.

### 2.3. Inclusion and Exclusion Criteria

Inclusion and exclusion criteria were defined according to the PCC framework. The selection aimed to include clinical, experimental, and preclinical studies relevant to bone regeneration through digital workflows and custom-milled bone grafts.

Inclusion criteria were as follows:

- Studies published in English between 2015 and 2025;
- Studies conducted on human subjects or validated animal models, involving digital technologies (CBCT, STL, CAD/CAM) for the design and fabrication of personalized bone grafts;
- Documented use of customized bone blocks (autologous, allogenic, xenogeneic, or synthetic);
- Studies clearly describing the digital workflow and surgical grafting into the recipient site;
- Clinical studies, cohort studies, case reports, case series, in vitro or in vivo experimental research.

Exclusion criteria were as follows:

- Articles not written in English;
- Studies without access to the full text;
- Narrative reviews, letters to the editor, conference abstracts;
- Studies involving 3D printing of surgical guides, splints, meshes, or membranes not associated with the fabrication of a solid personalized graft;
- Studies focused solely on prosthetics, implants, or CAD/CAM components with no bone regenerative purpose;
- Studies on bone regeneration using non-customized particulate grafts.

### 2.4. Study Selection

All articles identified through the search strategy were imported in RIS format into reference management and systematic screening software. The selection process was conducted in two phases:

(1) title and abstract screening for preliminary relevance assessment;
(2) full-text reading to evaluate eligibility based on the predefined criteria.

Two independent reviewers carried out the entire process. In case of disagreement, discussion with a third reviewer was held to reach a final decision. Reasons for exclusion at each phase were recorded and are reported in the PRISMA-ScR flow diagram.

### 2.5. Data Extraction

For each included article, data were extracted using a predefined grid designed to systematically and coherently summarize the information in alignment with the objectives of the scoping review. The following elements were collected from each study:

- Author and year of publication;
- Type of study (clinical, preclinical, in vitro, in vivo);
- Population (human or animal model);
- Type of bone defect treated (mandible or maxilla; vertical/horizontal/3D);
- Type of graft used (autologous, allogenic, xenogeneic, synthetic);
- Technologies used for the design and production of the graft (CBCT, STL segmentation, CAD modeling, CAM milling or 3D printing);
- Method of graft placement and type of fixation;
- Evaluated outcomes (e.g., bone integration, volumetric stability, complications, implant success);
- Main results, conclusions, and limitations as reported by the authors.

Data extraction was performed independently by two reviewers. In the case of discrepancies, the texts were re-examined until consensus was reached. Extracted data were organized in summary tables and analyzed through descriptive synthesis, in accordance with the aims of the scoping review. No meta-analysis was conducted, as it is not required by the PRISMA-ScR methodology.

Consistent with PRISMA-ScR guidelines, risk of bias assessment was not performed, as the purpose of this scoping review was to map the existing evidence rather than assess the methodological quality or comparative effectiveness of the included studies.

## 3. Results

### 3.1. Study Selection and Overview of Included Literature

The search strategy identified a total of 151 records: 62 from PubMed, 67 from Web of Science and 22 from Ovid MEDLINE. In addition to the database search, a manual screening of reference lists was performed to identify further studies meeting the inclusion criteria that had not been retrieved through the initial queries. This manual search involved reviewing the bibliographies of key reviews and primary studies identified during the full-text screening phase, as well as conducting targeted searches for foundational works frequently cited in the literature on digital workflows for bone regeneration. This supplementary process led to the inclusion of 6 additional studies. Although published before the defined date range (2015–2025), these articles were deemed highly relevant due to their early documentation of computer-assisted scaffold design and their significant contribution to the conceptual evolution of the CBCT-to-graft workflow.

After removing 44 duplicates, 107 articles remained for title and abstract screening.

Of these, 68 articles were excluded for the following reasons:

- 25—does not address personalized digital bone grafts in the oral/maxillofacial context.
- 17—digital workflow present, but no solid bone block/scaffold graft (e.g., planning, navigation, or generic CAD only).
- 10—3D printing general (biomaterials/platforms) not applied to personalized bone blocks or not oral.
- 9—prosthetic/guides/mesh focus only (no personalized bone block).
- 6—review article (excluded by protocol).
- 2—conventional GBR/particulate without personalization.

A total of 38 articles were retrieved for full-text assessment. Following full-text review, 21 additional articles were excluded:

- 3 involved non-biological synthetic scaffolds (e.g., polymers, PEEK, PLA) unrelated to CBCT-based milling workflows.
- 2 were purely in vitro studies without clinical or preclinical application.
- 6 addressed bone regeneration but outside the oral/maxillofacial region (orthopedic or cranial vault).
- 3 investigated conventional GBR without customization.
- 5 focused on digital guides only, not personalized blocks.
- 4 were reviews

As a result, 15 articles were included in the qualitative synthesis [11,12,13,15,16,18,19,20,21,22,23,24,25,26,27]. The papers included are summarized in Table 1.

A total of 15 articles were included in this scoping review, published between 2006 and 2025. Most of the studies consisted of case reports, case series, prospective observational studies, and experimental research on animal models. The most frequently represented countries of origin were Italy, Germany, the United States, South Korea, and Switzerland.

The studies focused on the regeneration of bone defects located in the mandible or maxilla, primarily involving atrophic posterior regions, edentulous ridges, and post-extraction defects. Most publications employed CBCT technology for defect segmentation, CAD or STL modeling of the bone block, and graft production via subtractive milling (CAM) or 3D printing. The materials used for graft fabrication included the following:

- Synthetic materials (e.g., hydroxyapatite, TCP, PLA): 13 articles.
- CAD/CAM-milled allogenic bone blocks: 7 articles.
- Customized xenografts: 1 articles, specifically a CAD/CAM-milled xenogeneic cancellous block designed from CBCT data.
- Unspecified or mixed materials: 2 articles.

None of the included studies described the complete workflow from CBCT to the milling of a customized biological block (autologous or xenogeneic) for grafting into a human patient. However, several studies involving synthetic scaffolds or allogenic blocks demonstrated the technical feasibility of anatomical customization. A comparative summary of quantitative metrics across included studies is provided in Table 2.

### 3.2. Characteristics and Technological Approaches of Included Studies

The 15 articles included in this scoping review were published between 2006 and 2024, reflecting almost two decades of progressive integration of digital technologies into personalized bone regeneration. The study designs were heterogeneous, comprising 7 case reports or case series [11,15,19,20,22,23,25], 5 prospective or retrospective clinical studies [16,18,21,24,26], 2 pilot randomized trials [20,24], 1 multicenter case series [27] and 1 preclinical animal study [19]. The most frequently represented countries of origin were Italy, Germany, and Egypt, with additional contributions from Austria, the Czech Republic, and the United States.

The bone defects investigated were located in both mandible and maxilla, with the majority involving posterior atrophic ridges [11,15,19,21,25], anterior maxillary deficiencies [16,22], or severe resorption requiring vertical augmentation [23,24,26]. Several studies addressed sinus lift procedures with digitally designed scaffolds [13,18], while others explored horizontal ridge augmentation with custom-milled blocks [20,21,26,27].

From a technological standpoint, all studies employed three-dimensional imaging, predominantly CBCT, as the initial diagnostic step for volumetric acquisition of the defect. Segmentation of CBCT data and conversion into STL files was consistently reported, followed by CAD-based customization of the graft design [11,12,13,15,16,18,19,20,21,22,23,24,25,26,27]. Subtractive manufacturing (milling) was the most common method of graft fabrication [11,15,16,20,21,22,23,24,25,26,27], while additive 3D printing was occasionally applied to synthetic or hydroxyapatite-based scaffolds [13,18,19].

Regarding graft materials, the included studies demonstrated a varied landscape:

- Customized allogeneic bone blocks were reported in 8 studies [11,15,16,20,21,22,23,26].
- Synthetic grafts (mainly hydroxyapatite scaffolds) were used in 3 studies [13,18,19].
- Customized xenogeneic blocks were evaluated in 1 multicenter case series [27].
- Autogenous bone in digitally guided interpositional grafting was employed in 1 prospective trial [24].
- Mixed materials or overviews including allo-, xeno-, and synthetic custom blocks were presented in 1 contribution [25].

Fixation was most commonly achieved with titanium screws [11,15,16,20,21,22,23,26,27], occasionally combined with resorbable membranes [16,22,26]. In sinus augmentation procedures, grafts were stabilized within the cavity without additional fixation [13,18]. Nazzal et al. [24] employed plates and screws for vertical interpositional grafts, while some reports did not provide detailed fixation protocols [19,25].

Clinical outcomes were heterogeneous: most case series and clinical studies reported successful implant placement with stable graft volume at short- to medium-term follow-up [11,15,16,20–23,25,26]. Histological or micro-CT validation was available only in a limited number of studies [15,22], showing viable bone formation within or around the grafted material. Blume et al. [16] provided volumetric 3D analysis, reporting a mean stability ratio of 67.8% at 6 months. Preclinical evaluation in beagle dogs demonstrated complete repair of critical-size bone defects with hydroxyapatite scaffolds combined with rhBMP-2 at 12 weeks [19].

The main limitations acknowledged across the included studies were the small sample size [11,15,20,22], lack of randomized comparisons or control groups [20,24], and restricted follow-up duration, often limited to ≤12 months [11,15,16,20,21,23]. Furthermore, although digital accuracy was frequently highlighted as a key advantage, only a minority of studies provided quantitative assessments of graft–host adaptation [16,19,22,26].

Among the included studies, digital workflows combining CBCT, STL file conversion, CAD modeling, and CAM fabrication were consistently reported [11,12,13,15,16,18,19,20,21,22,23,24,25,26,27]. Customized allogeneic blocks were most frequently described, followed by synthetic hydroxyapatite scaffolds, xenogeneic blocks, and digitally guided autogenous grafting. Fixation was mainly achieved with titanium screws, occasionally combined with membranes. Reported outcomes included successful graft adaptation, stable volumetric maintenance at follow-up, and histological evidence of vital bone in selected cases [15,16,22].

## 4. Discussion

This scoping review mapped the evolution of digital workflows for the design and fabrication of customized bone grafts in oral and maxillofacial surgery. Sixteen studies published between 2006 and 2024 were identified, demonstrating a gradual but fragmented adoption of CBCT-based planning, CAD modeling, and CAM fabrication techniques in bone regeneration [11,12,13,15,16,18,19,20,21,22,23,24,25,26,27].

The integration of three-dimensional imaging (CBCT), CAD modeling, and subtractive manufacturing (CAM) has paved the way for the development of customized bone grafts. CBCT represents a reliable technique for the three-dimensional assessment of bone defects, even when using low-dose protocols, proving effective in the identification and characterization of osseous lesions [28].

The earliest reports, such as Jacotti and Seemann, showed the feasibility of individualized scaffold design, albeit with limited case-based data [11,12]. In the following decade, Mangano and Figliuzzi provided pioneering evidence of synthetic hydroxyapatite scaffolds milled from CBCT data, primarily for sinus augmentation or vertical mandibular ridge reconstruction [13,18,19]. These studies confirmed the anatomical adaptability of custom grafts but were limited by the use of non-biological materials and small sample sizes.

Subsequent contributions by Schlee, Blume, Ambrosio, and Pfaffeneder-Mantai focused on allogeneic bone blocks manufactured through CAD/CAM milling [15,16,24,25]. These studies reported improved defect adaptation, reduced surgical time, and predictable volumetric stability, with short- to mid-term follow-ups confirming the feasibility of this approach in both single cases and multicenter prospective designs. Blume et al., for example, provided volumetric data demonstrating a stability ratio of 67.8% after six months, supporting the dimensional reliability of customized cancellous allogeneic blocks [16]. However, histological confirmation of new bone formation was scarce, with only Schlee and Hnítecka providing histological analyses confirming viable bone integration within the graft [15,27].

Other technological variations included the use of digitally guided autogenous grafts, as described by Nazzal et al., who demonstrated that computer-designed interpositional blocks enabled greater accuracy in vertical augmentation compared to conventional approaches [23]. Mounir and El Morsy investigated severely atrophic ridges using CAD/CAM-fabricated allogeneic blocks, with significant volumetric gains and implant survival at short-term follow-up, though both highlighted complications such as dehiscence in a subset of cases [20,21].

Xenogeneic CAD/CAM grafts remain underrepresented, with the multicenter series by Hnítecka representing the first histologically validated application in human patients, showing promising outcomes in terms of integration and volumetric stability [27].

Notably, only four of the sixteen included papers provided direct clinical documentation of customized CAD/CAM bone blocks (patient-specific design, milling, clinical implantation with clinical/radiographic outcomes) [16,21,25,26]. The remaining studies contributed complementary evidence on digital workflows, scaffold adaptation, or preclinical development.

Complications were explicitly reported in several of the included studies. Figliuzzi et al. described postoperative events such as delayed soft tissue healing following placement of a customized porous hydroxyapatite scaffold [19]. El Morsy et al. documented a single case of wound dehiscence during the healing period [21]. Blume et al. observed the partial resorption and dimensional reduction of the graft over time, though without compromising implant placement [16]. In the multicenter series by Hnítecka et al., isolated cases of membrane exposure and partial graft resorption were reported, with histology still confirming new bone formation in the majority of samples [27]. These findings indicate that, while customized grafts generally showed stable integration and supported implant placement, complications such as dehiscence, membrane exposure, or partial resorption remain possible and warrant careful surgical handling and follow-up.

The main methodological limitations across the included studies were small sample sizes, heterogeneity of patient populations, absence of control groups, and short follow-ups. In fact, most clinical studies reported follow-ups limited to ≤12 months, with very few extending beyond one year, which restricts conclusions on long-term stability and implant success. These issues limit the generalizability of findings and prevent robust comparative analyses. Moreover, the cost and technical expertise required for CAD/CAM graft design and production remain significant barriers to widespread adoption [16,19].

Despite these advances, none of the included studies reported the complete clinical workflow from CBCT acquisition through STL conversion, CAD, CAM milling, and clinical implantation of a biological autologous or xenogeneic block in human patients. This gap mirrors observations from systematic reviews outside the strict maxillofacial field, where synthetic or preclinical models dominate [29,30]. A central finding of this review is that, within the maxillofacial field, no clinical study has documented the entire “CBCT → STL → CAD → biological (autologous/xenogeneic) milling → clinical grafting” workflow in human patients, despite frequent reports of partial pathways (e.g., CBCT/CAD with synthetic prints, or CAD/CAM milling on allogenic blocks) [11,12,13,15,16,18,19,20,21,22,23,24,25,26,27].

Because graft–host congruence is expected to influence osteoconduction and mechanical stability, several studies emphasized precision of design and fit; when quantitatively reported, dimensional discrepancies were generally ≤0.5 mm and sometimes lower, aligning with clinically stable early outcomes [16,26]. From a biological standpoint, minimizing the gap between scaffold and bone is desirable; classic experimental work indicates that gaps > 0.3 mm can hinder predictable bone bridging (“jumping distance”), reinforcing the need for tight tolerances during digital design and milling [31]. Nonetheless, many accuracy assessments in the dental literature derive from digital superimpositions and radiographic metrics; histologic corroboration remains limited, which constrains biological interpretation beyond geometry [15,27].

Finally, upstream imaging parameters affect the fidelity of segmentation and downstream design. The accuracy of linear measurements on CBCT has been validated under appropriate protocols, but voxel size, artifacts, and HU calibration issues can influence measurement reliability and bone quality assessment—factors that must be standardized when translating CBCT data into CAD/CAM grafts [32,33,34].

This scoping review does not merely summarize existing literature but contributes a critical and updated perspective on the currently fragmented applications and technological disconnects that prevent full integration between virtual planning and biologically meaningful regenerative surgery. While the available evidence shows a clear direction toward personalized bone regeneration, it also underscores that no study has yet implemented the full digital-to-biological workflow in a validated clinical context. In this sense, our work offers a conceptual framework to guide future research toward the development of standardized, biologically validated, and clinically tested workflows, capable of making the paradigm of personalized bone regeneration a clinical reality. The increasing integration of imaging technologies and minimally invasive protocols in oral and maxillofacial surgery reflects a broader digital transformation in dentistry [35,36]. This growing interest in digital and biologically integrated solutions parallels trends in other areas of oral medicine, where advanced technologies have been effectively implemented for healing and pain management [37,38].

### 4.1. Future Research Directions

This review identified several important gaps and challenges that should guide future investigations in the field of personalized bone regeneration. First, although CBCT-based segmentation and CAD/CAM design are well described, no clinical study has yet documented the complete workflow from CBCT acquisition through STL conversion, CAD modeling, subtractive milling of biological grafts, and clinical implantation in human patients. This translational gap remains the most critical obstacle to clinical adoption. Second, current evidence is limited by small sample sizes, heterogeneous patient populations, and short follow-up durations, restricting the generalizability of outcomes. Large-scale, multicenter randomized controlled trials with standardized protocols are required to establish reproducible evidence. Third, while accuracy of graft fit is frequently reported with digital superimpositions, histological validation of bone integration is scarce, and should be systematically incorporated in both preclinical and clinical studies. Additionally, future research should prioritize the evaluation of biological materials suitable for CAD/CAM milling, particularly autologous and xenogeneic blocks, as their osteoinductive potential remains underexplored compared to synthetic and allogeneic alternatives. Finally, studies assessing cost-effectiveness, workflow efficiency, and long-term implant survival after digitally customized grafting are needed to translate these technologies from experimental applications to routine clinical use. Addressing these gaps will provide the methodological rigor and biological validation necessary to transform CBCT-based digital workflows into clinically predictable, patient-specific regenerative solutions.

### 4.2. Limitation

This scoping review presents several limitations. First, the literature search was conducted using three databases only (PubMed, Web of Science and Ovid MEDLINE), which—although comprehensive—may not capture all relevant studies available in the literature. Second, articles published in languages other than English and non–peer-reviewed publications (such as conference proceedings or theses) were excluded, potentially omitting useful data not accessible through conventional sources. Additionally, due to the descriptive nature of a scoping review, no quantitative synthesis was performed, nor was a risk of bias assessment conducted for the included studies. Another limitation of the available evidence is the scarce presence of histological validation and the absence of long-term follow-up data. Only a minority of studies included histological or micro-CT confirmation of new bone formation within or around the customized grafts, while most relied exclusively on radiographic or volumetric analyses. Moreover, the majority of clinical studies reported outcomes within 6–12 months, and only isolated cases extended observation up to 24 months. This restricts the possibility of drawing conclusions on the biological integration of the grafts and on the long-term stability of the reconstructed ridges and subsequent implant rehabilitation. Finally, the rapid evolution of digital technologies may cause some of the data presented to become outdated in a short period of time.

## 5. Conclusions

This scoping review systematically mapped the application of CBCT-based digital workflows in the design and fabrication of customized bone grafts for alveolar ridge augmentation. Sixteen studies published between 2006 and 2024 were included, showing increasing use of CAD/CAM technologies for allogeneic, xenogeneic, and synthetic blocks. Only four studies provided direct clinical documentation of customized bone blocks, and none reported the complete CBCT → STL → CAD → biological milling → clinical grafting workflow in human patients. The main gaps identified include limited histological validation, small sample sizes, short follow-ups, and a lack of randomized trials. Future research should prioritize standardized imaging and modeling protocols, biological validation of graft–host integration, and long-term clinical trials using autologous or xenogeneic blocks. Bridging these gaps will be essential to translate digital workflows into predictable, patient-specific regenerative solutions in oral and maxillofacial surgery.

## Figures and Tables

**Table 1:** Data extraction from selected studies.

Author and Year	Study Type	Model/Population	Bone Defect	Graft Material	Technology	Fixation	Main Outcomes	Conclusions/Limitations
Jacotti 2006 [11]	Case report	1 patient	Posterior mandible ridge deficiency	Customized allogeneic block	CBCT → CAD/CAM milling	Titanium screws + membrane	Feasible adaptation, implant placement achieved	Single case, short follow-up
Seemann 2010 [12]	Pilot clinical series	Patients (n not specified)	Edentulous maxilla	Individualized CAD/CAM synthetic scaffolds	CBCT → CAD/CAM	Screws	Feasibility; implant placement possible	Pilot; limited data
Mangano 2012 [13]	Case series	5 patients, 10 sinuses	Posterior maxilla, sinus lift	Custom HA blocks	CBCT-based CAD/CAM	Not specified	All implants functional at 2 years	Small sample, synthetic HA only
Schlee 2013 [15]	Case series	≤3 patients	Horizontal/vertical ridge defects	Allogeneic cancellous block	CBCT → CAD/CAM	Titanium screws + membrane	Histology showed vital bone, feasibility	Very small series
Mangano 2012 [18]	Clinical report	Patients (n not specified)	Maxillary sinus augmentation	Custom synthetic grafts	CBCT → CAD/CAM	Not specified	Successful augmentation, implants placed	Details incomplete
Figliuzzi 2013 [19]	Case report	1 patient	Posterior mandible, vertical defect	Porous HA scaffold	CBCT → CAD/CAM	Titanium screws	Implants placed at 6 months, functional	Limited evidence
Mounir 2019 [20]	Randomized clinical trial	16 patients, 32 implants	Severe ridge atrophy	Autograft/xenograft particulate with PEEK vs. Ti mesh	CAD/CAM PEEK vs. prebent Ti mesh	Screws	Comparable bone gain; PEEK easier handling	Pilot RCT, short-term
El Morsy 2020 [21]	Clinical study	14 patients, 34 implants	Anterior maxilla, severe atrophy	Autograft + xenograft particulate, PEEK containment	CAD/CAM PEEK sheets	Monocortical screws	Significant 3D bone gain; 1 dehiscence	No control, device study
Boogaard 2021 [22]	Case report	1 patient	Atrophic mandible, vertical defect	Customized allogeneic block (ACBB)	CBCT → CAD/CAM	Titanium screws	No resorption at 5 months, implant osseointegrated	Single case
Nazzal 2021 [23]	Pilot RCT	12 patients (6 guided vs. 6 conventional)	Vertical maxillary deficiency	Autogenous interpositional graft	3D-printed patient-specific guides	Plates/screws	Improved accuracy of vertical gain	Not a block, but relevant workflow
Pfaffeneder-Mantai 2022 [24]	Multicenter prospective	1 patient (case report in abstract)	Atrophic maxilla	Customized allogeneic blocks	CBCT → CAD/CAM	Screws	Stable augmentation, implants placed at 14 months	Case-based; limited data
Ambrosio 2023 [25]	Case series	2 patients	Horizontal ridge defects	Customized allogeneic blocks	CBCT → CAD/CAM	Likely screws + membrane	Reduced surgical time, stable augmentation, implants placed	Promising but very limited
Blume 2023 [16]	Prospective study	Patients with anterior maxillary atrophy	Anterior maxilla defects	Customized allogeneic cancellous block	CBCT → CAD/CAM; volumetric 3D analysis	Screws + membrane	Hard tissue gain 0.75 cm^3^ at 2 mo, 0.52 cm^3^ at 6 mo; stability ratio 67.8%	Reliable but small sample
Seidel 2024 [26]	Retrospective cohort	19 patients, 20 grafts	Horizontal/vertical defects	Allogeneic blocks (custom vs. prefabricated)	CBCT → CAD/CAM	Screws + membranes	Bone stability 87.6% vs. 83% at 6 mo; implant FU 44 mo	Retrospective, heterogeneous
Hnítecka 2024 [27]	Multicenter case series	Patients (n not specified)	Alveolar ridge defects	Customized xenogeneic blocks	CBCT → CAD/CAM	Screws + membrane	Feasible; some histology available	Sample size/outcomes limited

**Table 2:** Quantitative summary of included studies.

a					
**Author** **Year**	Jacotti 2006 [11]	Seemann 2010 [12]	Mangano 2012 [13]	Schlee 2013 [15]	Blume 2023 [16]
**Study type**	Case report Study type	Pilot series	Case series	Case series	Prospective study
**Population** **Model**	1 patient Population/Model	Patients (n NR)	5 pts/10 sinuses	≤3 patients	Patients with anterior maxillary atrophy
**Site/Defect**	Posterior mandible ridge deficiency Site/Defect	Edentulous maxilla	Posterior maxilla, sinus lift	Horizontal/vertical ridge defects	Anterior maxilla defects
**Graft material**	Customized allogeneic block Graft material	Synthetic individualized scaffold	Custom HA blocks	Customized allogeneic cancellous block	Customized allogeneic cancellous block
**Workflow**	CBCT → CAD/CAM milling Workflow	CBCT → CAD/CAM	CBCT → CAD/CAM	CBCT → CAD/CAM	CBCT → CAD/CAM + volumetric analysis
**Fit tolerance (mm)**	NR Fit tolerance (mm)	NR	NR	≤0.5 mm (qualitative)	≤0.5 mm
**Volumetric stability/Bone gain**	NR Volumetric stability/Bone gain	NR	All implants functional at 24 mo	NR	0.75 cm^3^ at 2 mo → 0.52 cm^3^ at 6 mo (67.8%)
**Follow-up (month)**	NR Follow-up	12	24	12	6
**Implant survival**	Implant placed (reported) Implant survival	NR	100% (10/10)	100% (reported)	NR
**Histology**	NR Histology	NR	NR	Yes	NR
**Notes**	Single case; short FU Notes	Feasibility pilot	Synthetic HA only	Very small series	Dimensional analysis
b					
**Author** **Year**	Mangano 2012 [18]	Figliuzzi 2013 [19]	Mounir 2019 [20]	El Morsy 2020 [21]	Boogaard 2021 [22]
**Study type**	Clinical report	Case report	RCT pilot	Clinical study	Case report
**Population** **Model**	Patients (n NR)	1 patient	16 pts/32 implants	14 pts/34 implants	1 patient
**Site/Defect**	Maxillary sinus augmentation	Posterior mandible, vertical defect	Severe ridge atrophy	Anterior maxilla, severe atrophy	Atrophic mandible, vertical defect
**Graft material**	Custom synthetic grafts	Porous HA scaffold	Auto/xeno particulate with PEEK vs. Ti mesh	Particulate + PEEK containment	Customized allogeneic block
**Workflow**	CBCT → CAD/CAM	CBCT → CAD/CAM	CAD/CAM PEEK vs. Ti mesh	CAD/CAM PEEK sheets	CBCT → CAD/CAM
**Fit tolerance (mm)**	NR	≤0.3 mm (qualitative)	NR	NR	NR
**Volumetric stability/Bone gain**	Successful augmentation; implants placed	Implants placed at 6 mo	Comparable bone gain	Vertical: 3.47 ± 1.46 mm; Horizontal: 3.42 ± 1.10 mm	No resorption at 5 mo
**Follow-up (month)**	NR	6	6	6	5
**Implant survival**	NR	100% (1/1)	NR	Implants placed	Implant osseointegrated
**Histology**	NR	Yes	NR	NR	Yes
**Notes**	Details incomplete	Functional outcome	Handling easier with PEEK	1 wound dehiscence	Single case
c					
**Author** **Year**	Nazzal 2021 [23]	Pfaffeneder-Mantai 2022 [24]	Ambrosio 2023 [25]	Seidel 2024 [26]	Hnítecka 2024 [27]
**Study type**	Pilot RCT	Prospective multicenter	Case series	Retrospective cohort	Multicenter case series
**Population** **Model**	12 pts (6 guided vs. 6 conv.)	Patients (case-based)	2 patients	19 pts/20 grafts	Patients (n NR)
**Site/Defect**	Vertical maxillary deficiency	Horizontal ridge defects	Horizontal ridge defects	Horizontal/vertical defects	Alveolar ridge defects
**Graft material**	Autogenous interpositional graft	Customized allogeneic blocks	Customized allogeneic blocks	Allogeneic blocks (custom vs. prefab)	Customized xenogeneic blocks
**Workflow**	3D-printed surgical guides	CBCT → CAD/CAM	CBCT → CAD/CAM	CBCT → CAD/CAM	CBCT → CAD/CAM
**Fit tolerance (mm)**	NR	NR	NR	≤0.5 mm	≤0.5 mm
**Volumetric stability/Bone gain**	Guided > conventional accuracy	Stable augmentation; implants placed	Stable augmentation; implants placed	Stability: 87.6% vs. 83% at 6 mo; MBL 0.41 mm	Volumetric stability; positive histology
**Follow-up (month)**	6	14	6	Up to 44	12
**Implant survival**	NR	NR	NR	100%	NR
**Histology**	NR	NR	NR	NR	Yes
**Notes**	Workflow study	Multicenter	Small case series	Heterogeneous	Early evidence

## Data Availability

No new data were created or analyzed in this study. Data sharing is not applicable to this article.

## Abbreviations

The following abbreviations are used in this manuscript:

β-TCP	β-Tricalcium Phosphate
CAD	Computer-Aided Design
CAM	Computer-Aided Manufacturing
CBCT	Cone-Beam computed tomography
GBR	Guided Bone Regeneration
HA	Hydroxyapatite
PLA	Polylactic Acid
PEEK	Polyether Ether Ketone
PRISMA-ScR	Preferred Reporting Items for Systematic Reviews and Meta-Analyses extension for Scoping Reviews
PCC	Population-Concept-Context
STL St	Standard Tessellation Language
